# The Changes in the Endothelial Function and Haemostatic and Inflammatory Parameters in Subclinical and Overt Hyperthyroidism

**DOI:** 10.1155/2013/981638

**Published:** 2013-12-03

**Authors:** Anna Popławska-Kita, Katarzyna Siewko, Beata Telejko, Anna Modzelewska, Janusz Myśliwiec, Robert Milewski, Maria Górska, Małgorzata Szelachowska

**Affiliations:** ^1^Department of Endocrinology, Diabetology and Internal Medicine, Medical University of Bialystok, Sklodowskiej-Curie 24A, 15-276 Białystok, Poland; ^2^Department of Statistics and Medical Informatics, Medical University of Bialystok, Szpitalna 37, 15-295 Białystok, Poland

## Abstract

*Introduction*. The aim of the present study was to compare the levels of circulating markers of endothelial function and low-grade inflammation in patients with subclinical and overt hyperthyroidism (OH) due to Graves disease (GD) and toxic nodular goiter (TNG). *Material and Methods*. The group studied consisted of 42 patients with GD, 75 patients with TNG, and 39 healthy controls. *Results*. Circulating markers of endothelial dysfunction were elevated in the patients with both SH and OH, but the concentrations of interleukin-12 (IL-12) (*P* < 0.05), IL-18 (*P* < 0.05), fibrinogen (*P* < 0.01), and von Willebrand factor (vWF) (*P* < 0.05) were significantly higher in the OH than in the SH group. The highest levels of IL-6, IL-12, IL-18, vWF, sVCAM-1, and fibrinogen were found in the patients with GD, but the differences between the GD, and TNG groups were not significant. In the subjects with OH serum IL-6 was positively associated with FT3 (*R* = 0.276, *P* < 0.05), FT4 (*R* = 0.273, *P* < 0.05), and thyroid peroxidase antibodies (*R* = 0.346, *P* < 0.01) levels. *Conclusion*. Our results may suggest that both SH and OH may be associated with endothelial dysfunction, which is reflected by decreased fibrinolytic activity, hypercoagulability, and increased levels of IL-6, IL-12, and IL-18 and depends not only on the cause but also on the degree of hyperthyroidism.

## 1. Introduction

Subclinical hyperthyroidism (SH) is characterised by suppressed serum thyroid-stimulating hormone (TSH) level, and free thyroxine (FT4) and triiodothyronine levels (FT3) remained within their reference ranges. SH is a common condition with the prevalence in the general population estimated as 0.6–16% [1–3] and can be diagnosed by thyroid function tests before the occurrence of clinical symptoms and complications [1]. Thyroid hormones can activate vascular endothelium and slow down the metabolism of adhesion molecules, thereby causing an elevation of their circulating levels. The hypothesis of vascular endothelium as a specific target for thyroid hormones is supported by enhanced endothelial function in hyperthyroidism [4]. It is commonly known that hyperthyroidism increases plasma levels of biologically active mediators involved in haemostasis, fibrinolysis, growth factors synthesis and the regulation of vessel tone, and permeability [5]. Endothelial dysfunction (ED) is characterized by an imbalance between relaxing and vasoconstricting factors, procoagulant and anticoagulant substances, and between proinflammatory mediators. Detection of ED is based on the assessment of endothelium-dependent vasomotion and on circulating markers of endothelial function (endothelin-1, von Willebrand factor [vWF], tissue plasminogen activator [tPA], plasminogen activator inhibitor-1 [PAI-1], and adhesion molecules) [6]. The soluble forms of various adhesion molecules have been demonstrated in various diseases, but their clinical significance is still undefined [4, 6]. Previous studies have found an association between SH and ED [7]; however, data regarding endothelium function in SH are insufficient. In the present study, we determined serum levels of the soluble forms of intercellular adhesion molecule-1 (sICAM-1), vascular cell adhesion molecule-1 (sVCAM-1), E-selectin, vWF, interleukin-6 (IL-6): IL-12, IL-18, and C-reactive protein (CRP) in patients with subclinical and overt hyperthyroidism (OH) due to Graves disease (GD) and toxic nodular goitre (TNG), in order to evaluate the possible role of these factors as potential markers of ED resulting from thyroid dysfunction. Thus, the aim of this study was to evaluate whether ED depends on the cause of thyrotoxicosis or on the degree of hyperthyroidism.

## 2. Material and Methods

One hundred and seventeen patients with thyroid dysfunction were included into the study: 42 patients (33 F/9 M) with GD and 75 patients (65 F/10 M) with TNG, as well as 39 euthyroid healthy controls of similar age and sex distribution. None of the participants received any antithyroid therapy. GD diagnosis was defined by hormonal hyperthyroidism, positive titres of antithyroid antibodies, and thyroid hypervascularity and hypoechogenicity on echography. Patients with GD met the following criteria: TSH <0.3 *μ*U/mL, the first onset of the disease, and no clinically apparent ophthalmopathy. In this group, 16 patients had SH and 26 patients had OH.

Patients with TNG presented as follows: nodular enlargement of the thyroid gland and no elevation of antithyroid antibodies and biochemical subclinical (45 patients) or overt (30 patients) hyperthyroidism. Subjects with diabetes, liver and kidney disorders, cardiac failure, acute or chronic infections, secondary causes of TSH suppression, and thyroid cancer, as well as these receiving any treatment influencing thyroid function, were excluded. All participants were nonsmokers. The blood samples were collected from antecubital vein, between 7:30 and 8:30 am, after an overnight fast, in order to avoid diurnal variations. Study participants were classified into 3 groups according to their thyroid function tests: subclinical hyperthyroidism (61 patients; 53 F/8 M), overt hyperthyroidism (56 patients; 45 F/11 M), and control group (CG) (39 patients; 26 F/13 M).

All subjects underwent a comprehensive assessment including documentation of medical history, physical examination, and laboratory tests. Physical examination included systolic and diastolic blood pressure and body weight and height measurements. All patients and controls gave informed consent to participate in the study before enrolment. The protocol was approved by the local ethics committee (Medical University of Bialystok).

Serum TSH (reference range 0.3–4.0 *μ*IU/mL), FT4 (reference range 0.71–1.85 ng/dL), and FT3 (reference range 1.45–3.48 pg/mL) concentrations were determined using enzyme immunoassays (Microparticle Enzyme Immunoassay—MEIA, Abbott Park, USA). Serum concentrations of sICAM-1, sVCAM-1, and E-selectin were determined by enzyme-linked immunoassays (Sandwich Enzyme Immunoassay, R&D Systems, USA). Serum levels of thyroid peroxidase antibodies (TPO-Ab) and thyroglobulin antibodies (TG-Ab) were determined by immunoassays (MEIA, Abbott AxSYM, USA). Serum concentrations of vWF and PAI-1 were also determined by commercial immunoassays (Asserachrom Diagnostica Stago, France). CRP was measured by Sandwich Enzyme Immunoassay (DSL-10-42100 Active, DSL, USA).

## 3. Statistical Analysis

STATISTICA 10.0 for Windows Software (StatSoft.Inc, Tulsa, USA) was used for the statistical analysis. Before analysis data were tested for normality of distribution using the Shapiro-Wilk test. Differences between the groups were compared by the Kruskal-Wallis test for multiple comparisons, and relationships between variables were tested using Spearman's rank correlation test. Categorical variables were compared by chi-square test. *P* value lower than 0.05 was considered statistically significant.

## 4. Results

The clinical characteristics of the patients with OH, SH, and normal thyroid function are shown in Table 1. As expected, the patients with OH are characterized by suppressed TSH and elevated FT3 and FT4 levels (by definition). Both groups with OH and SH had also significantly higher systolic blood pressure as compared with the controls (*P* < 0.05). Atrial fibrillation (AF) occurred in 16.4% of the patients with hyperthyroidism. Among the OH patients 19.64% had AF compared with 13.11% prevalence in the patients with SH (*P* < 0.05).

Serum concentrations of selected markers of inflammation and endothelial dysfunction in the SH and OH groups and in the control group are presented in Table 2. The patients with SH had significantly higher levels of IL-6 (*P* < 0.05), IL-12 (*P* < 0.05), PAI-1 (*P* < 0.01), and sVCAM (*P* < 0.001), whereas the subjects with SH were characterized by elevated concentrations of IL-6 (*P* < 0.05), Il-12 (*P* < 0.0001), IL-18 (*P* < 0.05), fibrinogen (*P* < 0.05), PAI-1 (*P* < 0.001), vWf (*P* < 0.0001), and sVCAM-1 (*P* < 0.0001) as compared with those of the control group. Moreover, the levels of IL-6 (*P* < 0.05), Il-18 (*P* < 0.05), fibrinogen (*P* < 0.01), and vWf (*P* < 0.05) in the OH patients were significantly higher than in the SH group. There were no significant differences in serum concentrations of CRP, E-selectin, and sICAM-1 in the patients with OH, SH, and normal thyroid function (Table 2).

The levels of selected markers of inflammation and endothelial dysfunction in the patients with hyperthyroidism due to GD and TNG and in the control group were compared in Table 3. The patients with GD had significantly higher levels of IL-6 (*P* < 0.001), IL-12 (*P* < 0.0001), IL-18 (*P* < 0.05), PAI-1 (*P* < 0.001), vWf (*P* < 0.0001), and sVCAM-1 (*P* < 0.0001), whereas the subjects with TNG were characterized by elevated concentrations of Il-12 (*P* < 0.001), PAI-1 (*P* < 0.01), vWf (*P* < 0.05), and sVCAM-1 (*P* < 0.001) in comparison with the controls. No significant differences in the serum levels of ED markers were observed between the hyperthyroid patients with GD and TNG (Table 3).

As shown in Table 4, in the OH group, TSH concentration negatively correlated with vWf and sVCAM-1 levels (*P* < 0.05), whereas FT3 concentration was positively associated with sICAM-1, sVCAM-1, vWf (all *P* < 0.01), fibrinogen and IL-6 levels (*P* < 0.05). In the same group, FT4 level correlated positively with IL-6 and fibrinogen concentrations (*P* < 0.05), whereas TPO-Ab concentration was positively associated with IL-6, E-selectin (*P* < 0.01), sVCAM-1, PAI-1, and vWf levels (*P* < 0.05). In the SH group, serum TSH level was negatively correlated with CRP, sICAM-1, and vWf concentrations (*P* < 0.05) (Table 5).

## 5. Discussion

Subclinical hyperthyroid disease is more common in women, in blacks, in the elderly, and in patients with low iodine intake. If SH is defined as thyroid state with serum TSH less than 0.4 *μ*IU/L, 3.2% of the population may be considered as having SH. If the diagnosis is limited to only those with serum TSH level lower than 0.1 *μ*IU/L, the prevalence of SH decreases to 0.7% [1]. Progression of SH to overt hyperthyroidism occurred at a rate of 5–8% per year [8]. The role of subclinical hyperthyroidism in cardiovascular disorders is a matter of debate and subject to ongoing controversy. It has been hypothesised that cardiovascular disorders associated with subclinical hyperthyroidism may be a direct effect of thyroid hormones excess [4, 7, 8]. Previous studies have demonstrated that when compared with age-matched control groups of euthyroid persons, patients with SH had a higher prevalence of arterial hypertension, a threefold increased risk for AF, dementia and Alzheimer's disease, and a significant increase in left ventricular mass, as well as a decreased bone mineral density and a fourfold increased risk for vertebral and hip fracture [9–11]. In our study, AF occurred in 16.4% of patients with hyperthyroidism and the occurrence of AF in the group of OH patients was significantly higher than that in the SH group (19.64% versus 13.11%). It has been also hypothesised that increased cardiovascular risk in SH patients may be related to significant changes in echocardiographic parameters [12], although Dörr et al. [13] found that SH had no impact on left ventricular mass index. Others authors have suggested that an associations of SH with cardiovascular disorders may result mainly from endothelial dysfunction [4, 7]; however, various disturbances of coagulation and fibrinolysis observed in patients with hyperthyroidism may range from subclinical laboratory abnormalities to clinically significant disorders of coagulation and rarely, major haemorrhage or thromboembolism [14]. According to the recent literature, most of the coagulation or fibrinolytic abnormalities associated with thyroid dysfunction are the consequences of the direct effects of thyroid hormones on the synthesis of various haemostatic factors. Hyperthyroidism is generally associated with hypercoagulability and decreased fibrinolytic activity [15]; however, hyperfibrinolysis was also reported [16] in those patients.

One of the most well-known markers of ED, which is synthesized and stored in endothelial Weibel-Palade bodies, seems to be vWF. In our study, serum vWF level was higher in the groups with both subclinical hyperthyroidism and overt hyperthyroidism, but the difference was significant only between the OH and control groups. In contrary to these findings, some authors found that the level of vWf was significantly higher in the SH group as compared with the healthy controls [4, 7]. In the present study, the highest vWF concentrations were noted in the patients with GD, which might reflect the possible influence of autoimmune and inflammatory processes on ED; however, the difference between the GD and TNG subjects was insignificant. Elevated vWf levels in the patients with OH were also reported by Myrup et al. [17] and Li et al. [18], whereas others found that vWF level was significantly increased in the subjects with hyperthyroidism in comparison with the control group, but no correlations with circulating FT3, FT4, and TSH were shown [19]. In the present study, vWf concentration correlated negatively with TSH level; however, no association with circulating thyroid hormones was observed. The next marker of ED dysfunction which was found to be elevated in the OH, but not in the SH group, was fibrinogen. The highest levels of this marker were noted in the patients with GD; however, the difference between the GD and TNG group was not significant. Similar results were obtained by other authors [19]. These findings may suggest exaggerated thrombo- and atherogenic processes in overt, but not in subclinical hyperthyroidism. It is yet to be clarified whether elevated fibrinogen level is a primary cause or a consequence of endothelial dysfunction. Some authors have showed that decreased serum TSH is an independent risk factor for elevated plasma fibrinogen level [20]. In our study, fibrinogen concentration did not correlate with TSH; however, in the patients with OH it was significantly and positively associated with both thyroid hormones. These results suggest that subjects with OH may be characterized by relative hypercoagulability and increased thromboembolic risk.

The imbalance between thrombotic and antithrombotic factors leads to pathological thrombus formation. Plasma plasminogen activator inhibitor-1 is the main inhibitor of the fibrinolytic system, and it is good evidence that higher plasma PAI-1 level may lead to the development of thromboembolytic complications [21] and cardiovascular disease [18, 22]. Both increased and decreased fibrinolytic activities have been reported in patients with hyperthyroidism. Elevated levels of PAI-1 antigen were found in overt and subclinical hyperthyroidism [23] and our study confirmed these results. There was no significant difference in PAI-1 levels between the GD and TNG patients, but we observed significant correlation between PAI-1 and TPO-Ab concentrations. Li et al. reported that serum thyroid hormone concentrations were strongly correlated with plasma PAI-1 [18], whereas Senturk et al. found that decreased serum TSH was an independent predictor of elevated PAI-1 antigen levels [23]. Contrary to these results, our study showed no significant correlations between PAI-1 and TSH, FT4 and FT3 concentrations.

Proinflammatory cytokine production has been previously studied in thyroid tissue from patients with GD, Hashimoto thyroiditis, and nontoxic goitre. These results demonstrated that the lymphocytic infiltrate found in autoimmune and nonautoimmune thyroid disorders is associated with enhanced cytokine production. It has been also shown that IL-6 promotes the changes in peripheral thyroid hormone metabolism, whereas IL-12 seems to be involved in the regulation of the central part of the hypothalamic-pituitary-thyroid axis during illness. IL-18 is a proinflammatory cytokine which shares important biological properties with IL-12, such as interferon gamma-inducing activity [23]. In the present study, we found elevated levels of these cytokines in the group with SH and in the groups with SH and OH. The highest concentrations of IL-6, IL-12, and IL-18 were observed in the subjects with GD, which might suggest a strong stimulation of the immune response; however, the difference in proinflammatory cytokine levels between the patients with hyperthyroidism due to GD and TNG was insignificant. It should be mentioned that previous measurements of IL6 in hyperthyroidism have shown conflicting results since both unchanged [23] and increased [24] levels of this cytokine have been reported. However, those measurements have been performed mostly in patients with autoimmune hyperthyroidism. It is worth noting that in the present study serum IL-6, IL-12, and IL-18 levels were found elevated not only in the GD but also in the TNG group, in which autoimmunity was not involved in the pathologic process. Moreover, although the cross-sectional design of our study limits any speculations about causal relationship between hyperthyroidism and increased cytokine production, a positive correlation between circulating IL-6 and both FT3 and FT4 might suggest that thyroid hormones directly modulate circulating markers of cell-mediated immune response. On the other hand, no association of serum IL-6 concentrations with changes in thyroid function was found by other authors [25]. In that study, serum IL-6 levels remained unchanged despite marked change in thyroid hormone levels, suggesting that increased IL-6 concentration reported in patients with GD was rather a result than a cause of autoimmune process underlying this disorder. In fact, it is well known that nonautoimmune thyroid disorders produce less striking changes in the immune response [26, 27].

On the basis of large-scale epidemiological and prospective hormone treatment trials, CRP has been reported to be a strong predictor of cardiovascular risk [28]. However, the impact of hyperthyroidism on CRP is still controversial. In the present study, CRP levels were slightly higher in the patients with SH and OH than in the healthy controls, but the difference was not significant.

Adhesion molecules play a central role in cell-to-cell communications for the ultimate induction of an effective immune response. ICAM-1 and VCAM-1 are coexpressed on activated endothelium in response to inflammatory mediators [29] Jublanc et al. found that autoimmune-mediated dysthyroidism was associated with increased peripheral blood concentrations of both sVCAM-1 and sICAM-1 [29, 30]. Also de Ciuceis et al. found that patients with GD had increased circulating levels of IL-18 and VCAM-1 [31]. Previously, it was reported that patients with GD showed a reduction in the physiological protective mechanisms against endothelial damage, probably induced by the increased inflammation and oxidative stress. Thus, thyroid hormones could activate vascular endothelium at nonrelated sites and prolong the metabolism of adhesion molecules, and thereby they cause an elevation of their circulating levels. In our study the level of sVCAM-1 was significantly higher in the GD and TNG groups with both OH and SH, but the concentrations of sICAM-1 and E-selectin did not differ markedly between the groups studied. In contrast to our results, Miyazaki et al. [32] found elevated levels of sELAM-l and sICAM-1 in the patients with GD; however, their expression in thyroid perifollicular endothelial cells was unchanged. It is also noteworthy that in the present study circulating sVCAM-1 and E-selectin correlated positively with TPO-Ab levels. These results seem to be in line with the findings of Graninger et al. [33], who reported that in contrast to other endothelial markers, which are elevated in nonimmunologically mediated hyperthyroidism, an elevation of sVCAM-1 could be specific for autoimmunological thyroid disorders. However, we should mention that in our study, sVCAM-1 levels were increased not only in the GD but also in the TNG patients and correlated negatively with TSH and positively with FT3 concentrations, which may suggest that elevated sVCAM-1 concentrations are dependent on autoimmune factors, as well as on the degree of hyperthyroidism. Moreover, we suggest that serum concentration of sVCAM but not sICAM or E-selectin, could be useful as a potential marker of disease activity in addition to thyroid hormones and antithyroid antibodies.

The main limitation of our study was the fact that the analysis was based on a single baseline determination but that may not reflect the patients' status over a long period. However, various methods used in the SH and OH groups for the treatment of hyperthyroidism, that is, antithyroid drugs, radioactive iodine therapy, and thyroid surgery, make any analysis concerning the effects of the treatment on the markers studied very difficult.

In conclusion, our results may suggest that both SH and OH may be associated with ED, which is reflected by decreased fibrinolytic activity, hypercoagulability, and increased levels of IL-6, IL-12, and IL-18 and depends not only on the cause but mainly on the degree of hyperthyroidism.

## Figures and Tables

**Table 1:** The clinical characteristics of the groups studied.

	Overt hyperthyroidism (*n* = 56) mean ± SD	Subclinical hyperthyroidism (*n* = 61) mean ± SD	Control group (*n* = 39) mean ± SD	*P* value
Sex F/M	45/11	53/8	26/13	NS
Age (years)	50.1 ± 12.8	48.6 ± 14.8	47.5 ± 11.8	NS
BMI (kg/m^2^)	24.6 ± 3.3	24.9 ± 3.6	25.1 ± 3.1	NS
HR (bpm)	92.0 ± 12.7	84.8 ± 7.6	73 ± 5.0	*P < 0.05, ***P < 0.05
Systolic pressure (mmHg)	142.8 ± 15.3	136.3 ± 14.4	126.2 ± 7.4	*P < 0.05, **P < 0.05
Diastolic pressure (mmHg)	82.4 ± 10.3	81.3 ± 8.3	77.8 ± 5.1	NS
TSH (uLU/mL)	0.024 ± 0.028	0.072 ± 0.052	1.161 ± 0.577	*P < 0.01, **P < 0.01
FT3 (pg/mL)	6.77 ± 4.5	2.74 ± 0.54	ne	***P < 0.01
FT4 (ng/dL)	3.74 ± 4.27	1.40 ± 0.30	ne	***P < 0.01

Differences between groups were compared by the Kruskal-Wallis test.

*Differences between OH and control groups, **differences between SH and control groups, ***differences between OH and SH, NS: not significant.

ne: not examined.

**Table 2:** Selected markers of inflammation and endothelial dysfunction in the patients with overt (OH) and subclinical hyperthyroidism (SH) and in the control group.

	OH (*n* = 56) median (C_25_–C_75_)	SH (*n* = 61) median (C_25_–C_75_)	Control (*n* = 39) median (C_25_–C_75_)	*P* value
IL-6 (ng/mL)	1.8 (1.1–2.9)	1.7 (1.1–3..8)	1.2 (0.7–1.8)	*P < 0.05, **P < 0.05
IL-12 (pg/mL)	3.1 (1.7–5.7)	1.8 (0.65–4.6)	0.5 (0.5–1.4)	*P < 0.0001, **P < 0.05, ***P < 0.05
IL-18 (pg/mL)	276.3 (186.0–368.5)	212.8 (161.0–300.2)	232.8 (181.1–257.8)	*P < 0.05, ***P < 0.05
CRP (ng/mL)	3.7 (3.1–5.4)	3.6 (2.7–4.7)	3.2 (2.7–4.3)	NS
Fibrinogen (mg/dL)	314.5 (253.5–374.0)	245.0 (172.0–303.0)	244.0 (185.0–325.0)	*P < 0.05, ***P < 0.01
PAI-1 (ng/mL)	54.6 (32.9–84.5)	59.3 (28.0–88.4)	31.5 (18.2–47.0)	*P < 0.001, **P < 0.01
vWF (ng/mL)	129.3 (117.0–144.4)	111.8 (987–132.5)	94.2 (73.0–116.3)	*P < 0.0001,***P < 0.05
E-selectin (ng/mL)	45.0 (34.0–68.0)	52.0 (36.0–70.0)	58.0 (40.0–68.0)	NS
sICAM-1 (ng/mL)	351.0 (294.0–409.0)	316.0 (262.0–378.0)	314.0 (278.0–404.0)	NS
sVCAM-1 (ng/mL)	1129.2 (943.0–1580.5)	1107.0 (835.0–1380.0)	792.0 (607.0–1010.0)	*P < 0.0001, **P < 0.001

Data are shown as medians with interquartile ranges.

Differences between groups were compared by the Kruskal-Wallis test.

*Differences between OH and control groups, **differences between SH and control groups, ***differences between OH and SH, NS: not significant.

**Table 3:** Selected markers of inflammation and endothelial dysfunction in the patients with Graves disease (GD), toxic nodular goitre (TNG), and in the control group.

	GD (*n* = 42) median (C_25_–C_75_)	TNG (*n* = 75) median (C_25_–C_75_)	Control group (*n* = 39) median (C_25_–C_75_)	*P* value
IL-6 (ng/mL)	2.3 (1.3–3.9)	1.5 (1.0–3.0)	1.2 (0.7–1.8)	*P < 0.001, *P* < 0.05
IL-12 (pg/mL)	3.3 (1.6–6.4)	1.9 (0.5–4.9)	0.5 (0.5–1.4)	*P < 0.0001, **P < 0.001
IL-18 (pg/mL)	287.2 (180.5–368.9)	217.9 (164.5–322.4)	232.8 (181.1–257.8)	*P < 0.05
PAI-1 (ng/mL)	58.5 (42.4–92.5)	56.4 (28.0–88.1)	31.5 (18.2–47.0)	*P < 0.001, **P < 0.01
vWF (ng/mL)	133.3 (118.7–141.8)	118.0 (98.7–135.9)	94.2 (73.0–116.3)	*P < 0.0001, **P < 0.05
E-selectin (ng/mL)	53.0 (42.0–80.0)	50.0 (34.0–66.0)	58.0 (40.0–68.0)	NS
sICAM-1 (ng/mL)	341.0 (300.0–418.0)	320.0 (258.0–394.0)	314.0 (278.0–404.0)	NS
sVCAM-1 (ng/mL)	1210.0 (950.0–1666.0)	1106.1 (840.0–1385.0)	792.0 (607.0–1010.0)	*P < 0.0001, **P < 0.001
CRP (ng/mL)	3.4 (2.9–4.7)	3.8 (2.9–4.9)	3.2 (2.7–4.3)	NS
Fibrinogen (ng/mL)	303.5 (212.0–368.0)	257.0 (181.0–358.0)	244.0 (185.0–325.0)	NS

Data are shown as medians with interquartile ranges.

Differences between groups were compared by the Kruskal-Wallis test.

*Differences between GD and control group, **differences between TNG and control group, NS: not significant.

**Table 4:** Spearman's rank correlation coefficients in the group with overt hyperthyroidism.

Correlation	Spearman's *R*	*P* value
TSH/sVCAM-1	−0.297	<0.05
TSH/vWF	−0.284	<0.05
FT3/sICAM-1	0.361	<0.01
FT3/sVCAM-1	0.346	<0.01
FT3/vWF	0.336	<0.01
FT3/IL-6	0.276	<0.05
FT3/Fibrinogen	0.275	<0.05
FT4/IL-6	0.273	<0.05
FT4/Fibrinogen	0.275	<0.05
TPO-Ab/sVCAM-1	0.273	<0.05
TPO-Ab/vWF	0.283	<0.05
TPO-Ab/IL-6	0.346	<0.01
TPO-Ab/E-selectin	0.401	<0.005
TPO-Ab/PAI-1	0.270	<0.05

**Table 5:** Spearman's rank correlation coefficients in the group with subclinical hyperthyroidism.

Correlation	Spearman's *R*	*P* value
TSH/sICAM-1	−0.275	<0.05
TSH/vWF	−0.314	<0.05
TSH/CRP	−0.279	<0.05
